# Estimation of finite population mean in a complex survey sampling

**DOI:** 10.1371/journal.pone.0324559

**Published:** 2025-05-28

**Authors:** Mohsin Abbas, Muhammad Ahmed Shehzad, Mahwish Rabia, Haris Khurram, Muhammad Ijaz

**Affiliations:** 1 Department of Statistics, Bahauddin Zakariya University, Multan, Pakistan; 2 Department of Statistics, Government College Women University Sialkot, Sialkot, Pakistan; 3 Department of Science and Humanities, National University of Computer and Emerging Sciences, Chiniot - Faisalabad Campus, Pakistan; 4 Department of Mathematics and Statistics, University of Haripur, Haripur, Pakistan; Dartmouth College Geisel School of Medicine, UNITED STATES OF AMERICA

## Abstract

Accurate estimation of the finite population mean is a fundamental challenge in survey sampling, especially when dealing with large or complex populations. Traditional methods like simple random sampling may not always provide reliable or efficient estimates in such cases. Motivated by this, the current study explores complex sampling techniques to improve the precision and accuracy of mean estimators. Specifically, we employ two-stage and three-stage cluster sampling methods to develop unbiased estimators for the finite population mean. Building upon these, the next phase of the study formulates unbiased mean estimators using stratified two- and three-stage cluster sampling. To further enhance the precision of these estimators, a ranked-set sampling strategy is applied to the secondary and tertiary sampling stages. Additionally, unbiased variance estimators corresponding to the proposed mean estimators are derived. Real-world datasets are utilized to demonstrate the application of these complex survey sampling methodologies, with results showing that the mean estimates derived using ranked set sampling are more accurate than those obtained via simple random sampling.

## 1 Introduction

In survey sampling, accurate and efficient estimation of population parameters, such as the population mean, is a critical challenge, especially when dealing with large, geographically dispersed populations. Conventional sampling techniques, like simple random sampling (SRS) and stratified random sampling, often prove inefficient in terms of cost and effort, particularly when faced with complex population structures. Cluster sampling, especially two-stage cluster sampling (2SCS) and three-stage cluster sampling (3SCS), provides a practical solution in these cases by enabling sampling of hierarchical populations. However, traditional estimators used in these methods frequently lack precision, particularly when population variability exists at different stages of sampling.

To address this issue, we propose new mean estimators and unbiased variance estimators under 2SCS and 3SCS, extending the approach to stratified two-stage cluster sampling (S2SCS) and stratified three-stage cluster sampling (S3SCS) schemes. Additionally, we incorporate ranked set sampling (RSS) at the secondary and tertiary stages of sampling to further improve estimator efficiency. RSS leverages ranking information to enhance the representativeness of the sample, which is particularly beneficial in stratified and multi-stage sampling designs. By deriving the mathematical formulas for mean, variance, and unbiased variance within these complex survey frameworks, we tackle the challenge of developing more efficient estimators that can effectively manage multi-level population structures.

Groups that arise naturally or are created by assembling the fundamental components of a limited population are known as clusters. For instance, villagers’ homes and their occupants. Cluster sampling involves the random selection of clusters from a population using a sampling strategy, followed by the collection of data from each unit in the selected cluster related to a research variable. When the sample frame of clusters is more easily accessible than that of the sampling units, cluster sampling can be used as an alternative to SRS. In these situations, the latter method is more effective in estimating population parameters. Cluster sampling, however, could limit how widely the sample units are used in the population. Increasing the number of clusters in the sample and then using a sampling technique to choose representative samples from the sampled clusters is one potential approach. Because this sampling technique consists of two stages, it is also known as 2SCS, with primary sampling units (PSUs) and secondary sampling units (SSUs) designating the first and second stages, respectively. The 3SCS, whose third-stage units are referred to as tertiary sampling units (TSUs), is a logical extension of the 2SCS. This plan is used to estimate the cost of inpatient treatment; hospitals are chosen in the first stage, wards are chosen in the second, and patients are chosen in the third stage. Moreover, in large-scale health and demographic surveys where populations exhibit both heterogeneity and wide geographical distribution, the 2SCS and 3SCS approaches can be combined with stratified random sampling to yield more representative samples. Variables used for stratification may encompass regions, urban-rural distinctions, topographical variations (e.g., plains vs. hills), or agro-climatic zones. For more details about 2SCS, 3SCS, and stratification, the readers may read [4,8,17,20] and the references cited therein.

RSS has proven useful in various real-world applications where exact measurements are expensive, but visual or comparative ranking is feasible; [22]. For instance, in forestry studies, researchers can estimate average tree height more efficiently by visually ranking trees and selecting a subset, thereby reducing costs and improving estimation accuracy. This method can be further enhanced using auxiliary variables, such as crown width, which is a strongly correlated with the variable of interest. Such auxiliary information may be derived from previous studies, field observations, sensor data, or existing datasets; see [2,5,24] for further details. In ecological assessments of hazardous waste sites, while accurate measurements might require costly radio-chemical methods, sites can often be visually ranked based on soil discoloration to estimate contamination levels.

In these situations, [18] suggested an RSS scheme as an efficient alternative to SRS. The RSS scheme gathers auxiliary information about the variable of interest in order to rank the selected sampling units, and thus helps in selecting more representative samples from the parent population; [13]. [31] established the statistical foundation for RSS by demonstrating the superiority of the mean estimator based on RSS over that based on SRS. [9] proved that even in the presence of ranking error, the RSS estimator of the population mean remains unbiased and is at least as efficient as the corresponding estimator under SRS.

Several advancements have been made in improving population mean estimators under RSS using auxiliary information. [15] enhanced the ratio estimator to estimate finite population mean under RSS originally proposed by [25]. [28] further examined the properties of a class of population mean estimators in RSS, building on the work of [32]. [2] utilized auxiliary information to estimate the finite population mean using ratio estimators under both SRS and median RSS. [5] introduced novel classes of estimators under RSS, while [6] proposed modified classes of such estimators. Additionally, [24] developed a regression estimator under RSS. These studies consistently show that estimators based on RSS are generally more precise and less biased than their counterparts under SRS.

Several advanced techniques have been proposed to enhance mean estimation under RSS. For instance, [3] developed mean and median estimators using a multistage RSS approach. [21] investigated a 2SCS method incorporating RSS (2SCS-RSS), where SRS and RSS are applied to the primary and secondary sampling units, respectively. [13] introduced a hybrid RSS scheme that integrates multiple existing RSS strategies to improve estimation accuracy. In subsequent research, [11] demonstrated that this hybrid RSS method, when employed within the 2SCS framework, can outperform the conventional 2SCS-RSS approach. For more comprehensive studies and further developments in mean estimation using RSS, refer to [1,12,33], and the references therein. Using a multistage RSS technique, [3] developed mean and median estimators. [21] examined population mean estimates based on 2SCS with RSS, or 2SCS-RSS, in which the primary and secondary sample frames, respectively, employed the SRS and RSS schemes. [13] developed the hybrid RSS scheme, which combines many current RSS techniques. This plan is more accurate than the mean estimator based on SRS and offers an impartial estimate of the population mean as well. [10] demonstrated in different research that the mean estimator based on the 2SCS with hybrid RSS scheme could be more accurate than the one based on the 2SCS-RSS scheme. Additional studies on mean estimation using RSS can be found in [1,12,33], and the references cited therein.

In the present study, we use the 2SCS and 3SCS methods to produce an unbiased estimator of the finite population mean. Furthermore, the RSS technique is employed in the 2SCS and 3SCS schemes’ secondary and tertiary sample frames, which are called 2SCS-RSS and 3SCS-RSS, respectively. Furthermore, the variances of these unbiased mean estimators are derived into precise mathematical formulas. Additionally, unbiased estimators of the mean estimators’ variances are also produced. The population mean is then estimated using the stratified 2SCS and 3SCS methods, or S2SCS and S3SCS, respectively, using SRS and RSS. The list of acronyms used throughout this study is provided in Table 1. The proposed estimators using RSS under complex sampling schemes solve two main problems: First, they increase precision by reducing the variance of the estimators, leading to more accurate population mean estimates. Second, they offer cost efficiency by using RSS to achieve higher precision without necessitating larger sample sizes. This makes the estimators highly suitable for large-scale surveys with financial or logistical constraints.

To demonstrate the practicality of these methods, we apply them to real-world datasets. This validation showcases the effectiveness of the proposed estimators under RSS in actual survey settings. Overall, the study presents a comprehensive framework for improving population mean estimation in complex survey designs, filling a significant gap in the literature by offering estimators that are both precise and computationally feasible in multi-stage sampling contexts.

Here is the remainder of the paper: A quick overview of the mean estimate using the SRS and RSS approaches is given in Sect 2. The population mean is computed using 2SCS/S2SCS in Sect 3 and 3SCS/S3SCS in Sect 4. In Sect 5, an empirical investigation is carried out. The discussion and conclusion of the paper, along with a summary of the key findings, are presented in Sect 6 and 7, respectively.

## 2 The mean estimation with SRS and RSS

The mean estimators based on SRS and RSS techniques, which are commonly employed in the parts that follow, are briefly reviewed in this section.

Let $\left(Y_{1},Y_{2},\ldots ,Y_{m}\right)$ represent a size-*m* random sample obtained by SRS without replacement from a finite population of *M* units with a mean, say $\mu_{Y}$. Let ${\bar{Y}}_{\text{SRS}}$ represent the mean estimate derived from this sample, as follows:

$${\bar{Y}}_{\text{SRS}}\;=\;\frac{1}{m}\sum_{j=1}^{m}Y_{j}$$ (1)

[8,29] shown that ${\bar{Y}}_{\text{SRS}}$ is an unbiased and strongly consistent estimator of $\mu_{Y}$. The variance of ${\bar{Y}}_{\text{SRS}}$ is given by:

$$V({\bar{Y}}_{\text{SRS}})\;=\;\frac{\lambda_{1}\sigma_{Y}^{2}}{m}\;,$$ (2)

where, $\lambda_{1}=(1-m/M)$, $\sigma_{Y}^{2}=\sum_{j=1}^{M}(Y_{j}-\mu_{Y})2/(M-1)$.

If we take sample using SRS with replacement then unbiased mean estimator, say ${\tilde{Y}}_{\text{SRS}}$ then its variance become:

$$V({\tilde{Y}}_{\text{SRS}})=\frac{\sigma_{Y}^{2}}{m}$$ (3)

RSS is a superior alternative to SRS when a small set of sampling units can be ranked, provided the ranking cost is negligible; [21]. This ranking can be done either visually with respect to the study variable or by using a low-cost but highly correlated auxiliary variable—for example, plant height as a proxy for crop yield in agricultural studies; [12]. The RSS works as follows: utilizing SRS with replacement from the underlying population, determine ${\text{k}}^{2}$ units. Next, divide these units into *k* sets at random, with *k* units in each set. Each set’s units are rated either visually or via a less expensive technique. Subsequently, for each value of *j* from 1,2,⋯,k, the unit with the *j*th smallest ranking is selected from the *j*th set of *k* units. This process completes the initial cycle of a ranked set sample of size *k*. To achieve a total sample size of *m* = *rk* units in a ranked set sample, the entire procedure can be iterated *r* times, resulting in *r* cycles.

In mathematical notation, let $\left(Y_{11l},Y_{12l},\ldots ,Y_{1kl}),(Y_{21l},Y_{22l},\ldots ,Y_{2kl}),\ldots ,(Y_{k1l},Y_{k2l},\ldots ,Y_{kkl}\right)$ be *k* separate random samples obtained from a finite population with mean $\mu_{Y}$ for the *l*th cycle, where l=1,2,…,r. For each *j*th sample $\left(Y_{j1l},Y_{j2l},\ldots ,Y_{jkl}\right)$, let $\left(Y_{j[1:k]l},Y_{j[2:k]l},\ldots ,Y_{j[k:k]l}\right)$ represent the judgment-order statistics with j=1,2,…,k. The RSS method was implemented to acquire $Y_{j[j:k]l}$ for j=1,2,…,k and l=1,2,…,r, resulting in a ranked set sample of size *rk*. Following the approach outlined in [30], an estimator of $\mu_{Y}$ using the RSS method, denoted as ${\bar{Y}}_{\text{RSS}}$, can be obtained as follows:

$${\bar{Y}}_{\text{RSS}}\;=\;\frac{1}{rk}\sum_{l=1}^{r}\sum_{j=1}^{k}Y_{j[j:k]l}$$ (4)

[31] showed that sample mean of ranked set sample is an unbiased estimator of $\mu_{Y}$ with its variance

$$V({\bar{Y}}_{\text{RSS}})=\frac{1}{rk}\left[\sigma_{Y}^{2}-\frac{1}{rk}\sum_{l=1}^{r}\sum_{j=1}^{k}(\mu_{Y[j:k]}-\mu_{Y})\right],$$ (5)

$$=\frac{\sigma_{Y}^{2}}{rk}-\frac{1}{rk^{2}}\sum_{j=1}^{k}(\mu_{Y[j:k]}-\mu_{Y})$$

$$=V({\tilde{Y}}_{\text{SRS}})-\frac{1}{rk^{2}}\sum_{j=1}^{k}(\mu_{Y[j:k]}-\mu_{Y}),$$ (6)

where $\mu_{Y[j:k]}$ is the mean of $Y_{j[j:k]l}$ and remain the same for all *r*. Furthermore, it can be clearly demonstrated that:

$$V({\tilde{Y}}_{\text{SRS}})\;=\;V({\bar{Y}}_{\text{RSS}})+\frac{1}{rk^{2}}\sum_{j=1}^{k}(\mu_{Y[j:k]}-\mu_{Y}).$$ (7)

[9] established the mathematical foundation of RSS and shown that ${\bar{Y}}_{\text{RSS}}$ is at least as efficient as ${\tilde{Y}}_{\text{SRS}}$ for the same sample size, as the variance $V({\bar{Y}}_{\text{RSS}})$ is always non-negative. In RSS, units are ranked within sets before selection, which ensures that each sample contains more representative information. This leads to lower variance in the estimates compared to SRS for the same sample size. As a result, RSS provides greater relative efficiency and reduces the number of observations required to achieve a desired level of accuracy, making it a powerful technique, especially when measurement costs are high or sample collection is resource-intensive. For more details on the computation of $V({\tilde{Y}}_{\text{RSS}})$ and $V({\tilde{Y}}_{\text{SRS}})$, the readers may see [10,12,21,30,31] and the references cited therein.

## 3 The mean estimation with 2SCS and S2SCS

In this section, we derive unbiased mean estimators for the 2SCS and S2SCS methods. Furthermore, the variances of these estimators’ unbiased estimators are also developed.

### 3.1 The mean estimation with 2SCS

In 2SCS, the population is divided into clusters, and a sample of clusters is drawn. Within each selected cluster, individual units are sampled. Assume that there are *N* PSUs in the target population *U*, and that for each i=1,2,⋯,N, the *i*th PSU includes *M*_*i*_ SSUs. Let *M*_*i*_ represent the total number of SSU in the *i*th PSU, and let *Y*_*i*,*j*_ represent the *j*th SSU that is present in the *i*th PSU, where $j=1,2,\cdots ,M_{i}$. The population mean, $\mu_{Y}$, under 2SCS may be expressed as

$$\mu_{Y}\;=\;\frac{1}{N\overset{―}{M}}\sum_{i=1}^{N}M_{i}\mu_{Y,i},$$ (8)

where,


$$\overset{―}{M}\;=\;\frac{1}{N}\sum_{i=1}^{N}M_{i}\;\text{and}\;\mu_{Y,i}\;=\;\frac{1}{M_{i}}\sum_{j=1}^{M_{i}}Y_{i,j}.$$

are the average cluster size and the mean computed from the *i*th PSU, respectively.

Estimating $\mu_{Y}$ under 2SCS-SRS and 2SCS-RSS schemes is our objective. Let $m_{i}=r_{i}k_{i}$ represent the number of SSUs chosen from the *i*th PSU and let *n* represent the number of PSUs chosen in the first step for this reason. The samples in both stages of the 2SCS-SRS scheme were selected using SRS without replacement. In contrast, the first stage of the 2SCS-RSS scheme also used SRS without replacement, while the second stage used RSS scheme.

We propose the following estimators for $\mu_{Y}$ under the 2SCS-SRS and 2SCS-RSS schemes:

$${\overset{―}{Y}}_{\text{SRS}}^{\text{2SCS}}=\frac{1}{n\overset{―}{M}}\sum_{i=1}^{n}M_{i}{\bar{y}}_{i,\text{SRS}}\;\text{and}$$ (9)

$${\overset{―}{Y}}_{\text{RSS}}^{\text{2SCS}}=\frac{1}{n\overset{―}{M}}\sum_{i=1}^{n}M_{i}{\bar{y}}_{i,\text{RSS}}\;,$$ (10)

respectively, where


$${\bar{y}}_{i,\text{SRS}}=\frac{1}{m_{i}}\sum_{j=1}^{m_{i}}Y_{i,j},\;\text{and}$$

$${\bar{y}}_{i,\text{RSS}}=\frac{1}{m_{i}}\sum_{l=1}^{r_{i}}\sum_{j=1}^{k_{i}}Y_{i,j[j:k_{i}]l}.$$

The following lemmas examine the properties of mean estimators, ${\overset{―}{Y}}_{\text{SRS}}^{\text{2SCS}}$ and ${\overset{―}{Y}}_{\text{RSS}}^{\text{2SCS}}$, under S2SCS-SRS and S2SCS-RSS schemes, respectively.

**Lemma 1:** ${\overset{―}{Y}}_{\text{SRS}}^{\text{2SCS}}$ and ${\overset{―}{Y}}_{\text{RSS}}^{\text{2SCS}}$ are unbiased estimators of $\mu_{Y}$.

**Proof.** By assigning indexes “1”, and “2” to represent the primary, and secondary stages of sampling, respectively, we can express it as follows:

$$E({\overset{―}{Y}}_{\text{SRS}}^{\text{2SCS}})=E_{1}\left[\frac{1}{n\overset{―}{M}}\sum_{i=1}^{n}M_{i}E_{2}({\bar{y}}_{i,\text{SRS}})\right]$$ (11)

$$=E_{1}\left[\frac{1}{n\overset{―}{M}}\sum_{i=1}^{n}M_{i}\mu_{Y,i}\right]$$

$$=\frac{1}{N\overset{―}{M}}\sum_{i=1}^{N}M_{i}\mu_{Y,i}=\mu_{Y}.$$

Note that $\left(M_{1}{\overset{―}{Y}}_{1},M_{2}{\overset{―}{Y}}_{2},\cdots ,M_{n}{\overset{―}{Y}}_{n}\right)$ may be observed as a simple random sample of size *n* from $\left(M_{1}{\overset{―}{Y}}_{1},M_{2}{\overset{―}{Y}}_{2},\cdots ,M_{n}{\overset{―}{Y}}_{N}\right)$ in the first steps of sampling. The unbiasedness of ${\overset{―}{Y}}_{\text{RSS}}^{\text{2SCS}}$ can be established following the approach in [21], thereby completing the proof.

**Lemma 2:** The variances of ${\overset{―}{Y}}_{\text{SRS}}^{\text{2SCS}}$ and ${\overset{―}{Y}}_{\text{RSS}}^{\text{2SCS}}$ are given by

$$V({\overset{―}{Y}}_{\text{SRS}}^{\text{2SCS}})=\frac{\lambda \sigma_{Y,b}^{2}}{n{\overset{―}{M}}^{2}}+\frac{1}{nN{\overset{―}{M}}^{2}}\sum_{i=1}^{N}\frac{\lambda_{i}M_{i}^{2}\sigma_{Y,i}^{2}}{m_{i}}\;\;\text{and}$$ (12)

$$V({\overset{―}{Y}}_{\text{RSS}}^{\text{2SCS}})=\frac{\lambda \sigma_{Y,b}^{2}}{n{\overset{―}{M}}^{2}}+\frac{1}{nN{\overset{―}{M}}^{2}}\sum_{i=1}^{N}\frac{M_{i}^{2}\sigma_{Y,i}^{2}}{m_{i}}-\frac{1}{nN{\overset{―}{M}}^{2}}\sum_{i=1}^{N}\frac{M_{i}^{2}}{k_{i}^{2}r_{i}}\sum_{j=1}^{k_{i}}{\left(\mu_{Y,i[j]}-\mu_{Y,i}\right)}^{2},$$

where,


$$\sigma_{Y,b}^{2}=\frac{1}{N-1}\sum_{i=1}^{N}(M_{i}\mu_{Y,i}-\overset{―}{M}\mu_{Y}{)}^{2},\;\sigma_{Y,i}^{2}\;=\;\frac{1}{M_{i}-1}\sum_{j=1}^{M_{i}}(Y_{i,j}-\mu_{Y,i}{)}^{2},$$

$$\lambda =\left(1-\frac{n}{N}\right),\;\text{and}\;\lambda_{i}\;=\;\left(1-\frac{m_{i}}{M_{i}}\right).$$

The term $\mu_{Y,i[j]}$ represents the expected value of $Y_{i,j[j:k_{i}]l}$ and remains constant across all cycles; see [10] for details.

**Proof.** We know that

$$V({\overset{―}{Y}}_{\text{SRS}}^{\text{2SCS}})=V_{1}[E_{2}({\overset{―}{Y}}_{\text{SRS}}^{\text{2SCS}})]+E_{1}[V_{2}({\overset{―}{Y}}_{\text{SRS}}^{\text{2SCS}})].$$ (13)

From Eq (11), $E_{2}({\overset{―}{Y}}_{\text{SRS}}^{\text{2SCS}})=\sum_{i=1}^{n}M_{i}\mu_{Y,i}/(n\overset{―}{M})$. Thus by using this result:

$$V_{1}[E_{2}({\overset{―}{Y}}_{\text{SRS}}^{\text{2SCS}})]=V_{1}\left(\frac{1}{n\overset{―}{M}}\sum_{i=1}^{n}M_{i}\mu_{Y,i}\right)=\frac{\lambda \sigma_{Y,b}^{2}}{n{\overset{―}{M}}^{2}},$$ (14)

$$E_{1}[V_{2}({\overset{―}{Y}}_{\text{SRS}}^{\text{2SCS}})]=E_{1}\left[\frac{1}{n^{2}{\overset{―}{M}}^{2}}\sum_{i=1}^{n}M_{i}^{2}.V_{2}({\bar{y}}_{i,\text{SRS}})\right]$$

$$=\frac{1}{nN{\overset{―}{M}}^{2}}\sum_{i=1}^{N}\frac{\lambda_{i}M_{i}^{2}\sigma_{Y,i}^{2}}{m_{i}}.$$ (15)

Eq (12) can be obtained by substituting Eq (14), and Eq (15) into Eq (13). Following a similar approach, one can derive the variance of ${\overset{―}{Y}}_{\text{RSS}}^{\text{2SCS}}$, thus concluding the proof. For more details about the derivation of ${\overset{―}{Y}}_{\text{RSS}}^{\text{2SCS}}$, the reader may see [11,21].

**Lemma 3:** Unbiased estimators of $V({\overset{―}{Y}}_{\text{SRS}}^{\text{2SCS}})$ and $V({\overset{―}{Y}}_{\text{RSS}}^{\text{2SCS}})$ are

$$\hat{V}({\overset{―}{Y}}_{\text{SRS}}^{\text{2SCS}})=\frac{\lambda {{\hat{\sigma }}^{2}}_{\text{SRS},Y,b}}{n{\overset{―}{M}}^{2}}+\frac{1}{nN{\overset{―}{M}}^{2}}\sum_{i=1}^{N}\frac{\lambda_{i}M_{i}^{2}{{\hat{\sigma }}^{2}}_{\text{SRS},Y,i}}{m_{i}-1},$$ (16)

and

$$\hat{V}({\overset{―}{Y}}_{\text{RSS}}^{\text{2SCS}})=\frac{\lambda {\hat{\sigma }}_{\text{RSS},Y,b}^{2}}{n{\overset{―}{M}}^{2}}+\frac{1}{2nN{\overset{―}{M}}^{2}}\sum_{i=1}^{n}\frac{M_{i}^{2}}{k_{i}^{2}r_{i}^{2}(r_{i}-1)}\sum_{l}\sum_{l^{{\;}^{\prime }}\neq l}\sum_{j}(Y_{i[j]l}-Y_{i[j]l^{{\;}^{\prime }}}{)}^{2},$$ (17)

respectively, where

$${\hat{\sigma }}_{\text{SRS},Y,b}^{2}=\frac{1}{n-1}\sum_{i=1}^{n}{\left(M_{i}{\bar{y}}_{i,\text{SRS}}-\overset{―}{M}\;{\overset{―}{Y}}_{\text{SRS}}^{\text{2SCS}}\right)}^{2},$$ (18)

$${\hat{\sigma }}_{\text{SRS},Y,i}^{2}=\frac{1}{m_{i}-1}\sum_{j=1}^{m_{i}}{\left(Y_{ij}-{\bar{y}}_{i,\text{SRS}}\right)}^{2},$$ (19)

$${\hat{\sigma }}_{\text{RSS},Y,b}^{2}=\frac{1}{n-1}\sum_{i=1}^{n}{\left(M_{i}{\bar{y}}_{i,\text{RSS}}-\overset{―}{M}\;{\overset{―}{Y}}_{\text{RSS}}^{\text{2SCS}}\right)}^{2}.$$ (20)

**Proof.** The proof of this lemma can be found in the S1 Appendix.

**Remark 1:** Assuming SRS with replacement is taken into account at the second-stage of sampling under 2SCS-SRS instead of SRS without replacement, then the variance of ${\tilde{Y}}_{\text{SRS}}^{\text{2SCS}}$ and its associated unbiased estimator are provided as follows:

$$V({\tilde{Y}}_{\text{SRS}}^{\text{2SCS}})=\frac{\lambda \sigma_{Y,b}^{2}}{n{\overset{―}{M}}^{2}}+\frac{1}{nN{\overset{―}{M}}^{2}}\sum_{i=1}^{N}\frac{M_{i}^{2}\sigma_{Y,i}^{2}}{m_{i}}\;\;\text{and}$$ (21)

$$\hat{V}({\tilde{Y}}_{\text{SRS}}^{\text{2SCS}})=\frac{\lambda {\hat{\sigma }}_{Y,b}^{2}}{n{\overset{―}{M}}^{2}}+\frac{1}{nN{\overset{―}{M}}^{2}}\sum_{i=1}^{n}\frac{M_{i}^{2}{\hat{\sigma }}_{Y,i}^{2}}{m_{i}-1},$$ (22)

respectively.

**Proof.** Following the approach of [8,12,21], the result can be readily derived using similar steps as in Lemmas 2 and 3.

As we seen above that RSS is more efficient than SRS with replacement because it improves the precision of the estimators by utilizing prior information about the population. Similarly, 2SCS-RSS is also more efficient than with 2SCS-SRS (with replacement) because RSS leverages prior ranking information to ensure more representative selections at each stage.

**Remark 2:** Under 2SCS scheme, $V({\overset{―}{Y}}_{\text{RSS}}^{\text{2SCS}})$ is more efficient than $V({\tilde{Y}}_{\text{SRS}}^{\text{2SCS}})$, i.e.

$$V({\tilde{Y}}_{\text{SRS}}^{\text{2SCS}})\;=\;V({\overset{―}{Y}}_{\text{RSS}}^{\text{2SCS}})+\frac{2}{n^{2}{\overset{―}{M}}^{2}}\;E_{1}\left[\sum_{i=1}^{n}\sum_{j<j^{\prime }=1}^{k_{i}}\sum_{l=1}^{r_{i}}\frac{M_{i}^{2}}{k_{i}^{2}r_{i}^{2}}(\mu_{Y,i[j]}-\mu_{Y,i}{)}^{2}\right].$$ (23)

This improves the precision of the estimators by reducing variance compared to 2SCS-SRS with replacement for the same sample size. Details of Remarks 1 and 2 can be found in [11,21].

Remark 2 demonstrate that RSS is more efficient than SRS with replacement by presenting the relevant mathematical expressions. This comparison shows that RSS reduces variance, improving the efficiency of the estimator under 2SCS. For more details, see [1,10,21], and the references cited therein.

### 3.2 The mean estimation with S2SCS

Assume that there are *L* strata in the target population, and that for each h=1,2,⋯,L, the size of the *h*th stratum comprises *N*_*h*_ units. Furthermore, the *h*th stratum has *N*_*h*_ PSUs, where the *i*th PSU for each of the values $i=1,2,\cdots ,N_{h}$, includes *M*_*i*,*h*_ SSUs. Let $j=1,2,\cdots ,M_{i,h}$ be the total number of SSUs inside the *i*th PSU, and let *Y*_*i*,*j*,*h*_ represent the *j*th SSU that is present in the *i*th PSU of the *h*th stratum. Under S2SCS, the finite population mean, $\mu_{Y}$, may be written as

$$\mu_{Y}\;=\;\sum_{h=1}^{L}W_{h}\mu_{Y,h}\;=\;\frac{1}{\sum_{h=1}^{L}N_{h}{\overset{―}{M}}_{h}}\sum_{h=1}^{L}N_{h}{\overset{―}{M}}_{h}\;\mu_{Y,h},$$ (24)

where


$$\mu_{Y,h}\;=\;\frac{1}{N_{h}{\overset{―}{M}}_{h}}\sum_{i=1}^{N_{h}}M_{i,h}\mu_{Y,i,h}\;\text{and}\;{\overset{―}{M}}_{h}=\frac{1}{N_{h}}\sum_{i=1}^{N_{h}}M_{i,h}$$

are the mean and average cluster size of the *h*th stratum, respectively.

A stratified two-stage cluster sample of size *n*_*h*_ is chosen from the *h*th stratum to estimate $\mu_{Y}$ using S2SCS. The sample sizes *n*_*h*_ are allotted according to an allocation system, such as Neyman allocation, proportional allocation, or equal allocation. In this case, two S2SCS variants—S2SCS-SRS and S2SCS-RSS—are taken into consideration. In the former case, the first and second stages sample are chosen using SRS without replacement, while in the latter case, SRS without replacement and RSS techniques are used to choose sample on first and second-stage, respectively. Under the S2SCS-SRS, we propose the following estimators to calculate $\mu_{Y}$:

$${\overset{―}{Y}}_{\text{SRS}}^{\text{S2SCS}}=\sum_{h=1}^{L}W_{h}{\overset{―}{Y}}_{\text{SRS,}h}^{\text{2SCS}},$$ (25)

where


$${\overset{―}{Y}}_{\text{SRS,}h}^{\text{2SCS}}=\frac{1}{n_{h}{\overset{―}{M}}_{h}}\sum_{i=1}^{n_{h}}M_{i,h}{\bar{y}}_{i,\text{SRS},h}$$

$$=\frac{1}{n_{h}{\overset{―}{M}}_{h}}\sum_{i=1}^{n_{h}}\frac{M_{i,h}}{m_{i,h}}\sum_{j=1}^{m_{i,h}}Y_{i,j,h}.$$

In the S2SCS-RSS method, the second sampling stage involves selecting a ranked set sample of size $m_{i,h}=r_{i,h}k_{i,h}$ from the *i*th PSU within the *h*th stratum. This sample is represented as $(Y_{i,j,h[j:k_{i,h}]l}$ where $j=1,2,\ldots ,k_{ij,h}$ and $l=1,2,\ldots ,r_{ij,h})$. Subsequently, an estimator for $\mu_{Y}$ under the S2SCS-RSS approach can be derived and given as follows:

$${\overset{―}{Y}}_{\text{RSS}}^{\text{S2SCS}}=\sum_{h=1}^{L}W_{h}{\overset{―}{Y}}_{\text{RSS,}h}^{\text{2SCS}},$$ (26)

where


$${\overset{―}{Y}}_{\text{RSS,}h}^{\text{2SCS}}=\frac{1}{n_{h}{\overset{―}{M}}_{h}}\sum_{i=1}^{n_{h}}M_{i,h}{\bar{y}}_{i,\text{RSS},h}$$

$$=\frac{1}{n_{h}{\overset{―}{M}}_{h}}\sum_{i=1}^{n_{h}}\frac{M_{i,h}}{m_{i,h}}\sum_{l=1}^{r_{hi}}\sum_{j=1}^{k_{i,h}}Y_{i,j[j:k_{i,h}]l}.$$

The mathematical derivation for 2SCS and S2SCS follows the same framework. The primary difference lies in S2SCS, where the estimation is performed independently within each stratum, and the overall estimate is obtained by aggregating results across strata. This approach ensures that the variability between strata is accounted for, leading to more precise population estimates.

The following lemmas examine the characteristics of mean estimators within S2SCS-SRS and S2SCS-RSS schemes.

**Lemma 4:** ${\overset{―}{Y}}_{\text{SRS}}^{\text{S2SCS}}$ and ${\overset{―}{Y}}_{\text{RSS}}^{\text{S2SCS}}$ are unbiased estimators of $\mu_{Y}$. The variances and their unbiased estimators for ${\overset{―}{Y}}_{\text{SRS}}^{\text{S2SCS}}$ and ${\overset{―}{Y}}_{\text{RSS}}^{\text{S2SCS}}$ are as follows:

$$V({\overset{―}{Y}}_{\text{SRS}}^{\text{S2SCS}})=\sum_{h=1}^{L}W_{h}^{2}\;V({\overset{―}{Y}}_{\text{SRS,}h}^{\text{2SCS}})\;\text{and}$$ (27)

$$V({\overset{―}{Y}}_{\text{RSS}}^{\text{S2SCS}})=\sum_{h=1}^{L}W_{h}^{2}\;V({\overset{―}{Y}}_{\text{RSS,}h}^{\text{2SCS}}),$$ (28)

and

$$\hat{V}({\overset{―}{Y}}_{\text{SRS}}^{\text{S2SCS}})=\sum_{h=1}^{L}W_{h}^{2}\;\hat{V}({\overset{―}{Y}}_{\text{SRS,}h}^{\text{2SCS}})\;\text{and}$$ (29)

$$\hat{V}({\overset{―}{Y}}_{\text{RSS}}^{\text{S2SCS}})=\sum_{h=1}^{L}W_{h}^{2}\;\hat{V}({\overset{―}{Y}}_{\text{RSS,}h}^{\text{2SCS}}),$$ (30)

respectively.

**Proof.** The Lemmas 1, 2, and 3 proofs are comparable to this one. The argument is completed by noting that the mathematical expressions of $V({\overset{―}{Y}}_{\text{SRS,}h}^{\text{2SCS}})$, $V({\overset{―}{Y}}_{\text{RSS,}h}^{\text{2SCS}})$, $\hat{V}({\overset{―}{Y}}_{\text{SRS,}h}^{\text{2SCS}})$ and $\hat{V}({\overset{―}{Y}}_{\text{RSS,}h}^{\text{2SCS}})$ are similar to $V({\overset{―}{Y}}_{\text{SRS}}^{\text{2SCS}})$, $V({\overset{―}{Y}}_{\text{RSS}}^{\text{2SCS}})$, $\hat{V}({\overset{―}{Y}}_{\text{SRS}}^{\text{2SCS}})$ and $\hat{V}({\overset{―}{Y}}_{\text{RSS}}^{\text{2SCS}})$, respectively, The only difference is that the former are calculated from the *h*th stratum for h=1,2,⋯,L.

**Remark 3:** Assuming SRS with replacement is taken into account at the secondary stage of sampling under S2SCS-SRS instead of SRS without replacement, then the variance of ${\tilde{Y}}_{\text{SRS}}^{\text{S2SCS}}$ and its associated unbiased estimator are provided as follows:

$$V({\tilde{Y}}_{\text{SRS}}^{\text{S2SCS}})=\sum_{h=1}^{L}W_{h}^{2}\;V({\tilde{Y}}_{\text{SRS},h}^{\text{2SCS}})\;\text{and}$$ (31)

$$\hat{V}({\tilde{Y}}_{\text{SRS}}^{\text{S2SCS}})=\sum_{h=1}^{L}W_{h}^{2}\;\hat{V}({\tilde{Y}}_{\text{SRS},h}^{\text{2SCS}}).$$ (32)

**Proof.** These proofs can be derived by following the same steps outlined in [12], where similar derivations were presented. The mathematical representation for $V({\tilde{Y}}_{\text{SRS},h}^{\text{2SCS}})$ and $\hat{V}({\tilde{Y}}_{\text{SRS},h}^{\text{2SCS}})$ closely resemble those of $V({\tilde{Y}}_{\text{SRS}}^{\text{2SCS}})$ and $\hat{V}({\tilde{Y}}_{\text{SRS}}^{\text{2SCS}})$, differing only in that the former are calculated from the *h*th stratum, thus completing the proof.

In 2SCS, $\sigma_{Y,b}^{2}$ denotes the between-cluster variance and $\sigma_{Y,i}^{2}$ the within-cluster variance. Both are used to compute the intra-cluster correlation coefficient (ICC), which measures similarity among units within clusters; [29]. In cluster sampling, it is generally assumed that observations within a cluster are correlated; [8]. To validate the assumption of intra-cluster correlation, ICCs were computed, which support the use of subsampling. When cluster sizes are unequal, the within-cluster variance $\sigma_{Y,i}^{2}$ varies across PSUs, so a pooled estimate ${\overset{―}{\sigma }}_{Y,i}^{2}$ is used, given by:


$${\overset{―}{\sigma }}_{Y,i}^{2}=\frac{\sum_{i=1}^{N}(M_{i}-1)\sigma_{Y,i}^{2}}{\sum_{i=1}^{N}(M_{i}-1)}.$$

The ICC is then computed as:


$$\rho_{2\text{SCS}}=\frac{\sigma_{Y,b}^{2}}{\sigma_{Y,b}^{2}+{\overset{―}{\sigma }}_{Y,i}^{2}}$$

In S2SCS, the ICC is computed similarly but separately within each stratum. For further details on pooled variance, refer to [16].

## 4 The mean estimation with 3SCS and S3SCS

In this section, we derive unbiased mean estimators for the 3SCS and S3SCS methods. Furthermore, the variances of these estimators’ unbiased estimators are also developed.

### 4.1 The mean estimation with 3SCS

In 3SCS, the samples are chosen in three stages: PSUs in the first stage, SSUs of the chosen PSUs in the second stage, and tertiary units from the chosen SSUs in the third stage. Similar to the 2SCS method, the SRS approach was employed to select samples across three stages. However, the RSS method can also be used for sample selection from a tertiary sampling frame. The 3SCS schemes are denoted as 3SCS-SRS and 3SCS-RSS, respectively. In the 3SCS-SRS scheme, samples were chosen using SRS without replacement in all three stages. Conversely, in the 3SCS-RSS scheme, SRS without replacement was used for the first two stages, whereas the RSS method was applied for sample selection in the third stage. The mean estimate derived from 3SCS-RSS is anticipated to be more accurate than that derived from 3SCS-SRS.

Assume that there are *N* PSUs in the target population, with *M*_*i*_ SSUs in each PSU and *T*_*ij*_ TSUs in each SSU. In the *j*th SSU of the *i*th PSU, where i=1,2,⋯,N, $j=1,2,\cdots ,M_{i}$, and $k=1,2,\cdots ,T_{ij}$, let *Y*_*ij*,*k*_ be the value for the *k*th TSU. Under 3SCS, the finite population mean, $\mu_{Y}$, may be written as

$$\mu_{Y}\;=\;\frac{1}{N\overset{―}{T}}\sum_{i=1}^{N}\sum_{j=1}^{M_{i}}T_{ij}\mu_{Y,ij},$$ (33)

where


$$\overset{―}{T}\;=\;\frac{1}{N}\sum_{i=1}^{N}\sum_{j=1}^{M_{i}}T_{ij}\;\text{and}\;\mu_{Y,ij}\;=\;\frac{1}{T_{ij}}\sum_{k=1}^{T_{ij}}Y_{ij,k}.$$

Here, $\mu_{Y,ij}$ be the mean computed from the *j*th SSU of the *i*th PSU.

We are interested in utilizing the 3SCS-SRS and 3SCS-RSS techniques to estimate $\mu_{Y}$. Let *n* represent the number of PSUs chosen in the first stage, *m*_*i*_ represents the number of SSUs chosen from the *i*th PSU, and *t*_*ij*_ signifies the count of tertiary units picked from the *j*th SSU. For 3SCS-SRS and 3SCS-RSS sampling schemes, we introduce the subsequent estimators to calculate $\mu_{Y}$:

$${\overset{―}{Y}}_{\text{SRS}}^{\text{3SCS}}=\frac{1}{n\overset{―}{T}}\sum_{i=1}^{n}\frac{M_{i}}{m_{i}}\sum_{j=1}^{m_{i}}T_{ij}{\bar{y}}_{ij,\text{SRS}}\;\text{and}$$ (34)

$${\overset{―}{Y}}_{\text{RSS}}^{\text{3SCS}}=\frac{1}{n\overset{―}{T}}\sum_{i=1}^{n}\frac{M_{i}}{m_{i}}\sum_{j=1}^{m_{i}}T_{ij}{\bar{y}}_{ij,\text{RSS}},$$ (35)

respectively, where


$${\bar{y}}_{ij,\text{SRS}}=\frac{1}{t_{ij}}\sum_{k=1}^{t_{ij}}Y_{ij,k}\;\text{and}\;{\bar{y}}_{ij,\text{RSS}}=\frac{1}{r_{ij}k_{ij}}\sum_{l=1}^{r_{ij}}\sum_{k=1}^{k_{ij}}Y_{ij,k[k:k_{ij}]l}.$$

The following lemmas examine the properties of ${\overset{―}{Y}}_{\text{SRS}}^{\text{3SCS}}$ and ${\overset{―}{Y}}_{\text{RSS}}^{\text{3SCS}}$ under 3SCS-SRS and 3SCS-RSS schemes, respectively.

**Lemma 5:** ${\overset{―}{Y}}_{\text{SRS}}^{\text{3SCS}}$ and ${\overset{―}{Y}}_{\text{RSS}}^{\text{3SCS}}$ are unbiased estimators of $\mu_{Y}$.

**Proof.** By assigning indexes “1”, “2”, and “3” to represent the primary, secondary, and tertiary stages of sampling, respectively, we can express it as follows


$$E({\overset{―}{Y}}_{\text{SRS}}^{\text{3SCS}})=E_{1}E_{2}\left[\frac{1}{n\overset{―}{T}}\sum_{i=1}^{n}\frac{M_{i}}{m_{i}}\sum_{j=1}^{m_{i}}T_{ij}E_{3}({\bar{y}}_{ij,\text{SRS}})\right]$$

$$=E_{1}E_{2}\left[\frac{1}{n\overset{―}{T}}\sum_{i=1}^{n}\frac{M_{i}}{m_{i}}\sum_{j=1}^{m_{i}}T_{ij}\mu_{Y,ij}\right]$$

$$=E_{1}\left[\frac{1}{n\overset{―}{T}}\sum_{i=1}^{n}\sum_{j=1}^{M_{i}}T_{ij}\mu_{Y,ij}\right]$$

$$=\frac{1}{N\overset{―}{T}}\sum_{i=1}^{N}\sum_{j=1}^{M_{i}}M_{ij}\mu_{Y,ij}=\mu_{Y}.$$ (36)

Using a similar approach, one can demonstrate that ${\overset{―}{Y}}_{\text{RSS}}^{\text{3SCS}}$ is unbiased, thus concluding the proof.

**Lemma 6:** The variances of ${\overset{―}{Y}}_{\text{SRS}}^{\text{3SCS}}$ and ${\overset{―}{Y}}_{\text{RSS}}^{\text{3SCS}}$ are given by

$$V({\overset{―}{Y}}_{\text{SRS}}^{\text{3SCS}})=\frac{\lambda \sigma_{Y,b}^{2}}{n{\overset{―}{T}}^{2}}+\frac{1}{nN{\overset{―}{T}}^{2}}\sum_{i=1}^{N}\frac{\lambda_{i}M_{i}^{2}\sigma_{Y,i}^{2}}{m_{i}}+\frac{1}{nN{\overset{―}{T}}^{2}}\sum_{i=1}^{N}\frac{M_{i}}{m_{i}}\sum_{j=1}^{M_{i}}\frac{T_{ij}^{2}\lambda_{ij}\sigma_{Y,ij}^{2}}{t_{ij}}\;\text{and}$$ (37)

$$V({\overset{―}{Y}}_{\text{RSS}}^{\text{3SCS}})=\frac{\lambda \sigma_{Y,b}^{2}}{n{\overset{―}{T}}^{2}}+\frac{1}{nN{\overset{―}{T}}^{2}}\sum_{i=1}^{N}\frac{\lambda_{i}M_{i}^{2}\sigma_{Y,i}^{2}}{m_{i}}+\frac{1}{nN{\overset{―}{T}}^{2}}\sum_{i=1}^{N}\frac{M_{i}}{m_{i}}\sum_{j=1}^{M_{i}}\frac{T_{ij}^{2}\sigma_{Y,ij}^{2}}{t_{ij}}\;\;-\frac{1}{nN{\overset{―}{T}}^{2}}\sum_{i=1}^{N}\frac{M_{i}}{m_{i}}\sum_{j=1}^{M_{i}}\frac{T_{ij}^{2}}{k_{ij}^{2}r_{ij}}\sum_{k=1}^{k_{ij}}(\mu_{Y,ij,k[k:k_{ij}]}-\mu_{Y,ij}{)}^{2},$$

respectively, where


$$\sigma_{Y,b}^{2}=\frac{1}{N-1}\sum_{i=1}^{N}(M_{i}\mu_{Y,i}-\overset{―}{M}\mu_{Y}{)}^{2},\;\sigma_{Y,i}^{2}=\frac{1}{M_{i}-1}\sum_{j=1}^{M_{i}}{\left(T_{ij}\mu_{Y,ij}-\mu_{Y,i}\right)}^{2}\;,$$

$$\sigma_{Y,ij}^{2}=\frac{1}{T_{ij}-1}\sum_{k=1}^{T_{ij}}{\left(Y_{ij,k}-\mu_{Y,ij}\right)}^{2}\;,\;\mu_{Y,i}=\frac{1}{M_{i}}\sum_{j=1}^{M_{i}}T_{ij}\mu_{Y,ij},\;\text{and}\;\lambda_{ij}=\left(1-\frac{t_{ij}}{T_{ij}}\right).$$

**Proof.** As it is known that

$$V({\overset{―}{Y}}_{\text{SRS}}^{\text{3SCS}})=E_{1}E_{2}V_{3}[{\overset{―}{Y}}_{\text{SRS}}^{\text{3SCS}}]+E_{1}V_{2}E_{3}[{\overset{―}{Y}}_{\text{SRS}}^{\text{3SCS}}]+V_{1}E_{2}E_{3}[{\overset{―}{Y}}_{\text{SRS}}^{\text{3SCS}}].$$ (38)

From Eq (36), we can write


$$V_{1}E_{2}E_{3}[{\overset{―}{Y}}_{\text{SRS}}^{\text{3SCS}}]=V_{1}E_{2}\left[\frac{1}{n\overset{―}{T}}\sum_{i=1}^{n}\frac{M_{i}}{m_{i}}\sum_{j=1}^{m_{i}}T_{ij}\mu_{Y,ij}\right]$$

$$=V_{1}\left(\frac{1}{n\overset{―}{T}}\sum_{i=1}^{n}M_{i}\mu_{Y,i}\right)=\frac{\lambda \sigma_{Y,b}^{2}}{n{\overset{―}{T}}^{2}}$$ (39)

and


$$E_{1}V_{2}E_{3}[{\overset{―}{Y}}_{\text{SRS}}^{\text{3SCS}}]=E_{1}V_{2}\left[\frac{1}{n\overset{―}{T}}\sum_{i=1}^{n}M_{i}{\bar{y}}_{i,\text{SRS}}\right]$$

$$=E_{1}\left[\frac{1}{n^{2}{\overset{―}{T}}^{2}}\sum_{i=1}^{n}M_{i}^{2}\;V_{2}({\bar{y}}_{i,\text{SRS}})\right]$$

$$=\frac{1}{nN{\overset{―}{T}}^{2}}\sum_{i=1}^{N}\frac{\lambda_{i}M_{i}^{2}\sigma_{Y,i}^{2}}{m_{i}},$$ (40)

where ${\bar{y}}_{i,\text{SRS}}=\sum_{j=1}^{m_{i}}T_{ij}\mu_{Y,ij}/m_{i}$. Also from Eq (36), we have


$$V_{3}[{\overset{―}{Y}}_{\text{SRS}}^{\text{3SCS}}]=\frac{1}{n^{2}{\overset{―}{T}}^{2}}\sum_{i=1}^{n}\frac{M_{i}^{2}}{m_{i}^{2}}\sum_{j=1}^{m_{i}}T_{ij}^{2}\;V_{3}({\bar{y}}_{ij,\text{SRS}})$$

$$=\frac{1}{n^{2}{\overset{―}{T}}^{2}}\sum_{i=1}^{n}\frac{M_{i}^{2}}{m_{i}^{2}}\sum_{j=1}^{m_{i}}\frac{T_{ij}^{2}\lambda_{ij}\sigma_{Y,ij}^{2}}{t_{ij}}.$$ (41)

Now take expectations of Eq (42) to get

$$E_{1}E_{2}V_{3}[{\overset{―}{Y}}_{\text{SRS}}^{\text{3SCS}}]\;=\;\frac{1}{nN{\overset{―}{T}}^{2}}\sum_{i=1}^{N}\frac{M_{i}}{m_{i}}\sum_{j=1}^{M_{i}}\frac{T_{ij}^{2}\lambda_{ij}\sigma_{Y,ij}^{2}}{t_{ij}}$$ (42)

Eq (37) can be obtained by substituting Eq (39), (40), and (42) into Eq (38). Following a similar approach, one can derive the variance of ${\overset{―}{Y}}_{\text{RSS}}^{\text{3SCS}}$, thus concluding the proof. The variance of ${\overset{―}{Y}}_{\text{RSS}}^{\text{3SCS}}$ can also be derived by following the same steps outlined in [12], where similar derivations were presented.

An unbiased estimator of variance is essential to ensure that the estimated variance accurately reflects the true variability of the population mean. Without unbiasedness, the variance estimator could systematically overestimate or underestimate the true variance, leading to misleading conclusions regarding the precision of the mean estimator. Accurate variance estimates are crucial for valid statistical inferences such as constructing confidence intervals and performing hypothesis tests. Using an unbiased variance estimator ensures that the reported uncertainty aligns with the actual sampling design, thus supporting more reliable decision making. Under the 3SCS-SRS and 3SCS-RSS, the unbiased estimator of the variance are given below.

**Lemma 7:** Unbiased estimators of $V({\overset{―}{Y}}_{\text{SRS}}^{\text{3SCS}})$ and $V({\overset{―}{Y}}_{\text{RSS}}^{\text{3SCS}})$ are

$$\hat{V}({\overset{―}{Y}}_{\text{SRS}}^{\text{3SCS}})=\frac{\lambda {{\hat{\sigma }}^{2}}_{\text{SRS},Y,b}}{n{\overset{―}{T}}^{2}}+\frac{1}{nN{\overset{―}{T}}^{2}}\sum_{i=1}^{n}\frac{\lambda_{i}M_{i}^{2}{\hat{\sigma }}_{\text{SRS},Y,i}^{2}}{m_{i}}+\frac{1}{nN{\overset{―}{T}}^{2}}\sum_{i=1}^{n}\frac{M_{i}}{m_{i}}\sum_{j=1}^{m_{i}}\frac{T_{ij}^{2}\lambda_{ij}{\hat{\sigma }}_{\text{SRS},Y,ij}^{2}}{t_{ij}-1}$$ (43)

and

$$\hat{V}({\overset{―}{Y}}_{\text{RSS}}^{\text{3SCS}})=\frac{\lambda {\hat{\sigma }}_{\text{RSS},Y,b}^{2}}{n{\overset{―}{T}}^{2}}+\frac{1}{nN{\overset{―}{T}}^{2}}\sum_{i=1}^{n}\frac{\lambda_{i}M_{i}^{2}{\hat{\sigma }}_{\text{RSS},Y,i}^{2}}{m_{i}}\;\;+\frac{1}{2nN{\overset{―}{T}}^{2}}\sum_{i=1}^{n}\frac{M_{i}}{m_{i}}\sum_{j=1}^{m_{i}}\frac{T_{ij}^{2}}{k_{ij}^{2}r_{ij}^{2}(r_{ij}-1)}\sum_{l}^{\;}\sum_{l^{{\;}^{\prime }}\neq l}^{\;}\sum_{k}^{\;}(Y_{ij,k[k:k_{ij}]l}-Y_{ij,k[k:k_{ij}]l^{{\;}^{\prime }}}{)}^{2},$$ (44)

respectively, where

$${\hat{\sigma }}_{\text{SRS},Y,b}^{2}=\frac{1}{n-1}\sum_{i=1}^{n}{\left(M_{i}{\bar{y}}_{i,\text{SRS}}-\overset{―}{T}\;{\overset{―}{Y}}_{\text{SRS}}^{\text{3SCS}}\right)}^{2},$$ (45)

$${\hat{\sigma }}_{\text{RSS},Y,b}^{2}=\frac{1}{n-1}\sum_{i=1}^{n}{\left(M_{i}{\bar{y}}_{i,\text{RSS}}-\overset{―}{T}\;{\overset{―}{Y}}_{\text{RSS}}^{\text{3SCS}}\right)}^{2},$$ (46)

$${\hat{\sigma }}_{\text{SRS},Y,i}^{2}=\frac{1}{m_{i}-1}\sum_{j=1}^{m_{i}}{\left(T_{ij}{\bar{y}}_{ij,\text{SRS}}-{\bar{y}}_{i,\text{SRS}}\right)}^{2},$$ (47)

$${\hat{\sigma }}_{\text{RSS},Y,i}^{2}=\frac{1}{m_{i}-1}\sum_{j=1}^{m_{i}}{\left(T_{ij}{\bar{y}}_{ij,\text{RSS}}-{\bar{y}}_{i,\text{RSS}}\right)}^{2},$$ (48)

$${\hat{\sigma }}_{\text{SRS},Y,ij}^{2}=\frac{1}{t_{ij}-1}\sum_{k=1}^{t_{ij}}{\left(Y_{ij,k}-{\bar{y}}_{ij,\text{SRS}}\right)}^{2}$$ (49)

**Proof:** The proof of this lemma can be found in the S1 Appendix.

**Remark 4:** The variance of ${\tilde{Y}}_{\text{SRS}}^{\text{3SCS}}$ and its unbiased estimators are provided if SRS with replacement is taken into consideration at the third stage of sampling instead of SRS without replacement, given by

$$V({\tilde{Y}}_{\text{SRS}}^{\text{3SCS}})=\frac{\lambda \sigma_{Y,b}^{2}}{n{\overset{―}{T}}^{2}}+\frac{1}{nN{\overset{―}{T}}^{2}}\sum_{i=1}^{N}\frac{\lambda_{i}M_{i}^{2}\sigma_{Y,i}^{2}}{m_{i}}+\frac{1}{nN{\overset{―}{T}}^{2}}\sum_{i=1}^{N}\frac{M_{i}}{m_{i}}\sum_{j=1}^{M_{i}}\frac{T_{ij}^{2}\sigma_{Y,ij}^{2}}{t_{ij}}\;,\;\text{and}$$ (50)

$$\hat{V}({\tilde{Y}}_{\text{SRS}}^{\text{3SCS}})=\frac{\lambda {\hat{\sigma }}_{\text{SRS},Y,b}^{2}}{n{\overset{―}{T}}^{2}}+\frac{1}{nN{\overset{―}{T}}^{2}}\sum_{i=1}^{n}\frac{\lambda_{i}M_{i}^{2}{\hat{\sigma }}_{\text{SRS},Y,i}^{2}}{m_{i}}+\frac{1}{nN{\overset{―}{T}}^{2}}\sum_{i=1}^{n}\frac{M_{i}}{m_{i}}\sum_{j=1}^{m_{i}}\frac{T_{ij}^{2}{\hat{\sigma }}_{\text{SRS},Y,ij}^{2}}{t_{ij}-1},$$ (51)

respectively.

**Proof.** By applying the same steps outlined in Lemmas 6 and 7, the result can be readily demonstrated. Additionally, these proofs can be derived following the approach in the remark of [21], where similar derivations were presented.

All mathematical expressions presented in the remarks and throughout the derivations are consistent with the framework of 2SCS/3SCS with SRS without replacement, reinforcing the generalizability of the approach. The primary goal of these remarks is to highlight that RSS offers a more efficient sampling strategy than SRS with replacement, justifying the focus on RSS for practical scenarios.

**Remark 5:** Under 3SCS scheme, $V({\overset{―}{Y}}_{\text{RSS}}^{\text{3SCS}})$ is more efficient than $V({\tilde{Y}}_{\text{SRS}}^{\text{3SCS}})$, i.e.

$$V({\tilde{Y}}_{\text{SRS}}^{\text{3SCS}})=V({\overset{―}{Y}}_{\text{RSS}}^{\text{3SCS}})+\frac{2}{n^{2}{\overset{―}{T}}^{2}}E_{1}E_{2}\left[\sum_{i=1}^{n}\frac{M_{i}^{2}}{m_{i}^{2}}\sum_{j=1}^{m_{i}}\frac{T_{ij}^{2}}{r_{ij}k_{ij}^{2}}\sum_{k<k^{\prime }=1}^{k_{ij}}(\mu_{Y,ij,k[k:k_{ij}]}-\mu_{Y,ij}{)}^{2}\right].$$ (52)

Similar to ${\overset{―}{Y}}_{\text{RSS}}^{\text{2SCS}}$, ${\overset{―}{Y}}_{\text{RSS}}^{\text{3SCS}}$ is at least as much efficient as ${\tilde{Y}}_{\text{SRS}}^{\text{3SCS}}$ when SRS with replacement is used at third stage of sampling because RSS leverages prior ranking information to ensure more representative selections at each stage. For more details about the computation, see [11], and [21].

### 4.2 The mean estimation with S3SCS

Assume that the target population is divided into *L* strata, with *N*_*h*_ units for each of the values of h=1,2,…,L included in each of the *h*th stratum’s size. Furthermore, the *h*th stratum has *N*_*h*_ PSUs, where the *i*th PSU for each of the values $i=1,2,\ldots ,N_{h}$ includes *M*_*i*,*h*_ SSUs. In addition, *T*_*ij*,*h*_ TSUs for $j=1,2,\ldots ,M_{i,h}.$ are contained in each SSU. For each $k=1,2,\ldots ,T_{ij,h}$, where *T*_*ij*,*h*_ is the total number of TSUs inside the *j*th SSU of the *i*th PSU, let *Y*_*ij*,*k*,*h*_ represent the *k*th TSU that is present in the *j*th SSU of the *i*th PSU within the *h*th stratum. The population mean under S3SCS may be expressed as

$$\mu_{Y}\;=\;\sum_{h=1}^{L}W_{h}\mu_{Y,h}\;=\;\frac{1}{\sum_{h=1}^{L}N_{h}{\overset{―}{T}}_{h}}\sum_{h=1}^{L}N_{h}{\overset{―}{T}}_{h}\;\mu_{Y,h},$$ (53)

where


$$\mu_{Y,h}\;=\;\frac{1}{N_{h}{\overset{―}{T}}_{h}}\sum_{i=1}^{N_{h}}\sum_{j=1}^{M_{i,h}}T_{ij,h}\mu_{Y,ij,h}\;\text{and}\;{\overset{―}{T}}_{h}=\frac{1}{N_{h}}\sum_{i=1}^{N_{h}}\sum_{j=1}^{M_{i,h}}T_{ij,h}$$

are the mean and average cluster size of the *h*th stratum, respectively.

A stratified three-stage cluster sample of size *n*_*h*_ is chosen from the *h*th stratum in order to estimate $\mu_{Y}$ with S3SCS. The sample size, *n*_*h*_, may be assigned according to an allocation strategy, such as proportional, equal, or Neyman allocation. Two S3SCS variations are taken into consideration here: S3SCS-SRS and S3SCS-RSS. In the former case, samples are chosen using SRS without replacement at all sampling stages, while in the latter case, SRS without replacement is used in the first two stages, with RSS used in the third stage. We take into consideration the following population mean estimator under the S3SCS-SRS:

$${\overset{―}{Y}}_{\text{SRS}}^{\text{S3SCS}}=\sum_{h=1}^{L}W_{h}{\overset{―}{Y}}_{\text{SRS,}h}^{\text{3SCS}},$$ (54)

where


$${\overset{―}{Y}}_{\text{SRS,}h}^{\text{3SCS}}=\frac{1}{n_{h}{\overset{―}{T}}_{h}}\sum_{i=1}^{n_{h}}\frac{M_{i,h}}{m_{i,h}}\sum_{j=1}^{m_{i,h}}T_{ij,h}{\bar{y}}_{ij,\text{SRS},h}$$

$$=\frac{1}{n_{h}{\overset{―}{T}}_{h}}\sum_{i=1}^{n_{h}}\frac{M_{i,h}}{m_{i,h}}\sum_{j=1}^{m_{i,h}}\frac{T_{ij,h}}{t_{ij,h}}\sum_{k=1}^{t_{ij,h}}Y_{ij,k,h}.$$

In the S3SCS-RSS method, the third sampling stage involves selecting a ranked set sample of size $t_{ij,h}=r_{ij,h}k_{ij,h}$ from the *j*th SSU of the *i*th PSU within the *h*th stratum. This sample is represented as $(Y_{ij,k,h[k:k_{ij,h}]l}$ where $k=1,2,\ldots ,t_{ij,h}$ and $l=1,2,\ldots ,r_{ij,h})$. Subsequently, an estimator for $\mu_{Y}$ under the S3SCS-RSS approach can be derived.

$${\overset{―}{Y}}_{\text{RSS}}^{\text{S3SCS}}=\sum_{h=1}^{L}W_{h}{\overset{―}{Y}}_{\text{RSS,}h}^{\text{3SCS}},$$ (55)

where


$${\overset{―}{Y}}_{\text{RSS,}h}^{\text{3SCS}}=\frac{1}{n_{h}{\overset{―}{T}}_{h}}\sum_{i=1}^{n_{h}}\frac{M_{i,h}}{m_{i,h}}\sum_{j=1}^{m_{i,h}}T_{ij,h}{\bar{y}}_{ij,\text{RSS},h},$$

$$=\frac{1}{n_{h}{\overset{―}{T}}_{h}}\sum_{i=1}^{n_{h}}\frac{M_{i,h}}{m_{i,h}}\sum_{j=1}^{m_{i,h}}\frac{T_{ij,h}}{r_{ij,h}k_{ij,h}}\sum_{l=1}^{r_{ij,h}}\sum_{k=1}^{k_{ij,h}}Y_{ij,k[k:k_{ij,h}]l}.$$

The following lemmas examine the characteristics of mean estimators within 3SCS-SRS, and 3SCS-RSS schemes.

**Lemma 8:** Both ${\overset{―}{Y}}_{\text{SRS}}^{\text{S3SCS}}$ and ${\overset{―}{Y}}_{\text{RSS}}^{\text{S3SCS}}$ are unbiased estimators of $\mu_{Y}$; see [12] for a similar derivation. The variances and their unbiased estimators for ${\overset{―}{Y}}_{\text{SRS}}^{\text{S3SCS}}$ and ${\overset{―}{Y}}_{\text{RSS}}^{\text{S3SCS}}$ are as follows:

$$V({\overset{―}{Y}}_{\text{SRS}}^{\text{S3SCS}})=\sum_{h=1}^{L}W_{h}^{2}\;V({\overset{―}{Y}}_{\text{SRS,}h}^{\text{3SCS}})\;\text{and}$$ (56)

$$V({\overset{―}{Y}}_{\text{RSS}}^{\text{S3SCS}})=\sum_{h=1}^{L}W_{h}^{2}\;V({\overset{―}{Y}}_{\text{RSS,}h}^{\text{3SCS}}),$$ (57)

and

$$\hat{V}({\overset{―}{Y}}_{\text{SRS}}^{\text{S3SCS}})=\sum_{h=1}^{L}W_{h}^{2}\;\hat{V}({\overset{―}{Y}}_{\text{SRS,}h}^{\text{3SCS}})\;\text{and}$$ (58)

$$\hat{V}({\overset{―}{Y}}_{\text{RSS}}^{\text{S3SCS}})=\sum_{h=1}^{L}W_{h}^{2}\;\hat{V}({\overset{―}{Y}}_{\text{RSS,}h}^{\text{3SCS}}).$$ (59)

**Proof.** The result follows from Lemmas 5, 6, and 7 and can also be derived using the approach in the lemmas of [12]. The argument is completed by noting that the mathematical representation of $V({\overset{―}{Y}}_{\text{SRS,}h}^{\text{3SCS}})$, $V({\overset{―}{Y}}_{\text{RSS,}h}^{\text{3SCS}})$, $\hat{V}({\overset{―}{Y}}_{\text{SRS,}h}^{\text{3SCS}})$ and $\hat{V}({\overset{―}{Y}}_{\text{RSS,}h}^{\text{3SCS}})$ are similar to $V({\overset{―}{Y}}_{\text{SRS}}^{\text{3SCS}})$, $V({\overset{―}{Y}}_{\text{RSS}}^{\text{3SCS}})$, $\hat{V}({\overset{―}{Y}}_{\text{SRS}}^{\text{3SCS}})$ and $\hat{V}({\overset{―}{Y}}_{\text{RSS}}^{\text{3SCS}})$, respectively. The only difference is that the former are calculated from the *h*th stratum for h=1,2,⋯,L, which completes the proof.

**Remark 6:** Assuming SRS with replacement is taken into account at the tertiary stage of sampling under S3SCS-SRS instead of SRS without replacement, then the associated variance and its unbiased estimator are provided as follows:

$$V({\tilde{Y}}_{\text{SRS}}^{\text{S3SCS}})=\sum_{h=1}^{L}W_{h}^{2}\;V({\tilde{Y}}_{\text{SRS,}h}^{\text{3SCS}})\;\text{and}$$ (60)

$$\hat{V}({\tilde{Y}}_{\text{SRS}}^{\text{S3SCS}})=\sum_{h=1}^{L}W_{h}^{2}\;\hat{V}({\tilde{Y}}_{\text{SRS,}h}^{\text{3SCS}})$$ (61)

**Proof.** The result can be demonstrated by following the steps outlined in Lemmas 6 and 7, as well as the approach in [12]. The mathematical representation for $V({\tilde{Y}}_{\text{SRS,}h}^{\text{3SCS}})$ and $\hat{V}({\tilde{Y}}_{\text{SRS,}h}^{\text{3SCS}})$ closely resemble those of $V({\tilde{Y}}_{\text{SRS}}^{\text{3SCS}})$ and $\hat{V}({\tilde{Y}}_{\text{SRS}}^{\text{3SCS}})$, differing only in that the former are calculated from the *h*th stratum, thus completing the proof.

In 3SCS, $\sigma_{Y,b}^{2}$, $\sigma_{Y,i}^{2}$, and $\sigma_{Y,ij}^{2}$ represent the between-cluster, between-secondary unit, and within-tertiary unit variances, respectively. For unequal cluster sizes, the ICC is computed using pooled variance estimates as follows:


$${\overset{―}{\sigma }}_{Y,i}^{2}=\frac{\sum_{i=1}^{N}(M_{i}-1)\sigma_{Y,i}^{2}}{\sum_{i=1}^{N}(M_{i}-1)},\;{\overset{―}{\sigma }}_{ij}^{2}=\frac{\sum_{i=1}^{N}\sum_{j=1}^{M_{i}}(T_{ij}-1)\sigma_{ij}^{2}}{\sum_{i=1}^{N}\sum_{j=1}^{M_{i}}(T_{ij}-1)}$$

The ICC from 3SCS is then computed as:


$$\rho_{3\text{SCS}}=\frac{\sigma_{Y,b}^{2}}{\sigma_{Y,b}^{2}+{\overset{―}{\sigma }}_{Y,i}^{2}+{\overset{―}{\sigma }}_{Y,ij}^{2}}$$

In S3SCS, ICC is computed similarly but separately within each stratum. The ICC computed from each population is provided in the corresponding description.

## 5 Empirical study

A comparison of the relative efficiencies (REs) of the mean estimators under RSS over SRS using 2SCS/3SCS, and S2SCS/S3SCS is presented in this section.

### 5.1 Population I

A dataset sourced from the Centers for Disease Control, is associated with the Second National Health and Nutrition Examination Survey (NHANES-II). It comprises 10,351 units, representing the entire non-institutionalized civilian population of the United States, including all 50 states and the District of Columbia. The data is separated into four geographic regions (REGs): midwestern, southern, northeastern, and western, each farther subdivided into specific locations (LOCs). With a success probability of 0.50, random numbers are generated from a Bernoulli distribution to stratify the dataset into two strata, with “0” indicating Stratum-I, and “1” indicating Stratum-II. This data is available at [7] and can also be found in the study conducted by [19]. This dataset is also provided in the Supporting Information section under S3 Files. In this study, body mass index (BMI) is taken as study variable. In the 2SCS/S2SCS design, REG served as the PSU and BMI as the SSU, while in the 3SCS/S3SCS design, REG, LOC, and BMI were designated as PSU, SSU, and TSUs, respectively. The ICCs computed from this population are: 0.9988 for 2SCS, 0.9995 for S2SCS, 0.9835 for 3SCS, and 0.9224 for S3SCS, indicating strong within-cluster homogeneity.

### 5.2 Population II

The dataset utilized in this study originates from [26] and can also be accessed online at [27], where it has been uploaded to Figshare repository for public availability. A cross-sectional investigation was conducted in Multan District, a prominent region in southern Punjab, Pakistan, from January to March 2020. Spanning $3721\;{\text{km}}^{2}$, Multan District is the most populous (4.7 million inhabitants) and largest urban center in southern Punjab. According to the Pakistan Bureau of Statistics’ 2017 data, the district had 350,000 children and adolescents aged 3-18 years. Study participants were required to meet the following criteria: be between 3 and 18 years old, attend school regularly, have no chronic illnesses, be free from physical or mental disabilities, and be willing to take part in the research. The complete dataset comprises 1040 units, categorized into two strata: Stratum-I (males) and Stratum-II (females). BMI is considered the study variable, denoted as *Y*, in this study. For the 2SCS/S2SCS design, socioeconomic status (SES) and BMI are treated as PSU and SSU, respectively. In the 3SCS/S3SCS design, SES, Age, and BMI are considered as PSU, SSU, and TSUs, respectively. The ICCs computed from this population are 0.9999 (2SCS), 0.9999 (S2SCS), 0.9900 (3SCS), and 0.9892 (S3SCS).

### 5.3 Population III

In addition, another dataset is based on the Social & Household Integrated Economic Survey, which was carried out in Pakistan between 2013 and 2014. This dataset (that contains 46257 households) comprises all rural and urban areas of Pakistan’s four provinces, namely Punjab, Khyber Pakhtunkhwa, Sindh, and Balochistan, while excluding FATA and military restricted areas. This dataset of either rural or urban area is first partitioned into provinces, where each province is further divided into various enumeration blocks (EBs). Whilst the rural and urban areas are two strata. This dataset can be found in [14] and also available online at [23] where it has been uploaded to Figshare repository for public availability. In this study, the income of a household (HH) is considered the study variable, denoted as *Y*. The province and yearly total income of an HH are designated as the PSU, and SSU for 2SCS, and S2SCS. In the case of 3SCS, and S3SCS, the province, EB, and yearly total income of an HH are identified as the PSU, SSU, and TSU, respectively. The ICCs—0.9999 for 2SCS, S2SCS, 3SCS, and S3SCS—indicate exceptionally high within-cluster homogeneity, supporting the use of sub-sampling method.

### 5.4 The RE of mean estimators based on RSS over SRS without replacement

In this section, the RE of the mean estimators derived from aforementioned complex survey sampling schemes are calculated using the previously described datasets, varying the values of *n*, *m*_*i*_, and $\left(r_{i},k_{i}\right)$. We chose RE as the primary criterion for evaluating our estimators due to its practical relevance and ease of interpretation in complex sampling designs. Focused on cluster sampling frameworks, RE enabled us to directly compare the efficiency of proposed estimators using real-world data without relying on additional assumptions that may not apply in complex survey situations.

In order to calculate the variances of the mean estimators, 10,000 samples are taken from each population using a specific sampling plan. The following formulas are used to estimate the variances of mean estimators derived from 2SCS-RSS, and 2SCS-SRS:


$$V({\overset{―}{Y}}_{\text{SRS}}^{\text{2SCS}})\approx \frac{1}{\left(\psi -1\right)}\;\sum_{i=1}^{\psi }({\overset{―}{Y}}_{\text{SRS},i}^{\text{2SCS}}-\mu_{Y}{)}^{2},$$

$$V({\overset{―}{Y}}_{\text{RSS}}^{\text{2SCS}})\approx \frac{1}{\left(\psi -1\right)}\;\sum_{i=1}^{\psi }({\overset{―}{Y}}_{\text{RSS,}i}^{\text{2SCS}}-\mu_{Y}{)}^{2},$$

respectively, where ψ=10,000. The same is true for mean estimators derived from other sampling schemes as well. The RE of ${\overset{―}{Y}}_{\text{RSS}}^{\text{2SCS}}$ regarding ${\overset{―}{Y}}_{\text{SRS}}^{\text{2SCS}}$ is given by,


$$\text{RE}({\overset{―}{Y}}_{\text{RSS}}^{\text{2SCS}},{\overset{―}{Y}}_{\text{SRS}}^{\text{2SCS}})\;=\;\frac{V({\overset{―}{Y}}_{\text{SRS}}^{\text{2SCS}})}{V({\overset{―}{Y}}_{\text{RSS}}^{\text{2SCS}})}.$$

As a similar approach, it may be possible to calculate the REs of other mean estimators that use S2SCS, 3SCS, and S3SCS schemes.

**Fig 1:**
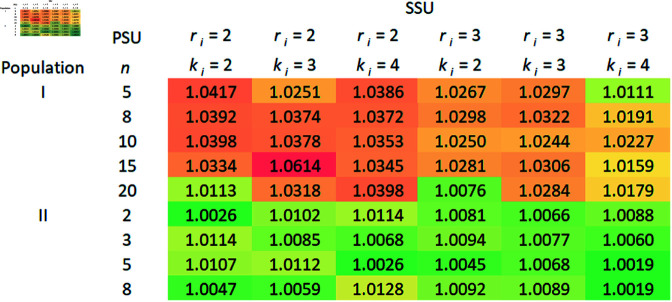
2SCS-RSS vs 2SCS-SRS (without replacement).

**Fig 2:**
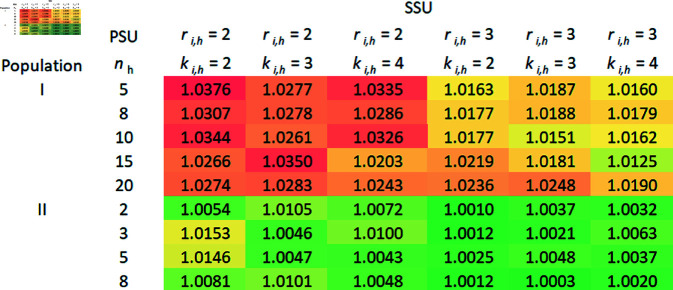
S2SCS-RSS vs S2SCS-SRS (without replacement).

**Fig 3:**
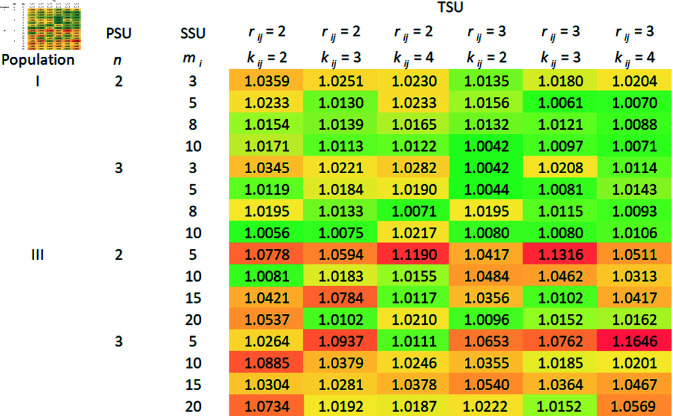
3SCS-RSS vs 3SCS-SRS (without replacement).

**Fig 4:**
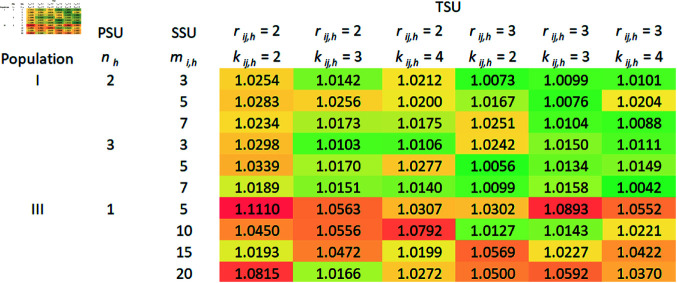
S3SCS-RSS vs S3SCS-SRS (without replacement).

Theoretically, we have seen that RSS is more efficient than SRS with replacement. In order to determine the efficiency of RSS over SRS without replacement, we compare the RE of mean estimators derived from SRS without replacement with those from RSS under complex survey schemes, and the RE is calculated. Tables 1–4 given in S2 Tables, display the RE for mean estimators using 2SCS/S2SCS with SRS without replacement, compared to 2SCS-RSS/S2SCS-RSS. Similarly, Tables 1–4 show the RE for mean estimators derived from 3SCS/S3SCS with SRS without replacement in relation to 3SCS-RSS/S3SCS-RSS. Notably, in all sampling frames—primary, secondary, and tertiary—both with, and without stratification, RSS-based mean estimators demonstrated slightly higher efficiency than those from SRS without replacement, as all RE values were above one. Moreover, the effect of increasing cycles, in secondary or tertiary sampling frame under 2SCS and 3SCS, on the RSS method remains unclear. Generally, REs increase with larger sample sizes at the primary, secondary, or tertiary stages of sampling. However, the RSS with small cycle size is more precise than the large number of cycles as we can see in Figs 1, 2, 3, and 4. In addition to the gain in efficiency observed with the RSS method compared with SRS without replacement, our proposed method consistently outperformed in terms of RE across all the complex survey sampling schemes discussed above. The RSS-based estimators performed well in both 2SCS and 3SCS, regardless of the sampling frame’s stratification. Thus, RSS provides a reliable alternative to SRS for the estimation of population mean in these complex survey sampling schemes.

Graphical representations of the RE of mean estimators based on the RSS scheme over SRS without replacement under complex survey sampling schemes were created, as illustrated in Figs 1, 2, 3, and 4. Across the different schemes, Population I consistently exhibits higher efficiency ratios compared to Populations II and III, demonstrating that the RSS method performs significantly better than SRS without replacement for Population I. Efficiency tends to increase with specific combinations of PSUs, SSUs, and TSUs, with the highest REs observed under moderate PSU sizes. For instance, in the 3SCS scheme, Population III displays greater variability, with maximum efficiency values occurring at smaller PSU sizes, specifically with *r*_*ij*_ = 3 and *k*_*ij*_ = 4. The maximum gain in efficiency in this scenario is 16%. In contrast, Population II shows relatively stable and lower efficiency values across all configurations, indicating minimal improvements from RSS compared to SRS without replacement for this population. Overall, the results emphasize the potential for RSS to significantly outperform SRS without replacement in specific scenarios, particularly in more complex sampling schemes and for populations that exhibit greater variability in characteristics.

In the next section, we discuss the computational complexity of our proposed method including its limitation and practical implication of these estimators under complex survey sampling.

## 6 Discussion

The computational complexity of the proposed method primarily depends on the number of stages in the sampling process (2SCS or 3SCS), the inclusion of stratification, and the type of sampling method (RSS, SRS with replacement or SRS without replacement). In 2SCS, the complexity arises from selecting a sample of PSU in the first stage and sampling units from each selected cluster in the second stage. When you introduce stratification, additional computational resources are required to determine strata and then apply the same sampling procedure within each stratum, which increases the overall complexity due to separate estimations within each stratum. In 3SCS, the complexity increases further due to an additional level of sampling within sub-clusters in the third stage, where the computational effort scales with the number of clusters and sub-clusters. When applying RSS at the final stage, under 2SCS or 3SCS, the process requires extra steps for ranking the units before selecting the sample, adding computational load due to the ranking operations. SRS with replacement or without replacement requires less computation compared to RSS, but it still requires tracking the random selection of units across stages. In short, the computational complexity of the proposed methods increases with the number of stages (from two-stage to three-stage), the inclusion of stratification, and the use of RSS, which introduces additional ranking steps and calculations. The simplest case would involve 2SCS with SRS, while the most complex scenario would involve S3SCS with RSS at the final stage.

The choice between 2SCS and 3SCS schemes depends on the population structure, available resources, and desired precision. 2SCS is ideal when the population is divided into primary clusters (e.g., schools or villages) with low intra-cluster variability, making it cost-effective and simpler by sampling more units within fewer clusters. In contrast, 3SCS is suitable for populations with further subdivisions (e.g., schools into classes and then students) and high intra-cluster variability, offering detailed sampling and better representation of smaller sub-clusters. Although more resource-intensive, it provides higher precision by capturing sub-cluster variability. Hence, 2SCS is practical for limited resources or homogeneous clusters, while 3SCS is preferable for greater accuracy in complex population structures.

The ICCs computed across survey sampling schemes confirmed the presence of correlation among units within clusters. This supports the underlying assumption of intra-cluster dependence, which is critical to the effectiveness of sub-sampling. By validating this assumption empirically, the study strengthens the theoretical foundation of the proposed estimators under complex sampling designs.

The proposed estimators are highly practical for large-scale surveys and studies with complex populations, such as in health, education, or environmental research. They efficiently capture variability across multiple levels, enhancing precision in 2SCS and 3SCS designs. This makes them ideal for large surveys, where simpler sampling methods would be inefficient or costly. The use of RSS is further improves accuracy, especially in fields like agriculture. However, the increased complexity, particularly in three-stage or stratified designs, requires more resources and expertise, limiting their feasibility for smaller studies. While these methods enhance precision and efficiency, they may not be ideal for all situations due to their complexity, resource demands, and sensitivity to assumptions like homogeneity and non-response.

Regarding alternative estimators, our focus was to provide a robust assessment of the RE for the proposed estimators within the context of complex survey sampling, leveraging auxiliary information to improve efficiency. Incorporating multiple alternative estimators, though valuable, was beyond the scope of this study due to computational considerations and our focus on empirical performance with real datasets. Moreover, we did not compare our estimators to the Cramér–Rao bound because it may not be directly applicable to multistage sampling due to its complex dependence structures. Furthermore, the Cramér–Rao bound is typically relevant in scenarios that assume independent and identically distributed samples, unlike the correlated structure found in cluster sampling.

## 7 Conclusion

In this study, we have developed mean estimators for two-stage cluster sampling (2SCS) and three-stage cluster sampling (3SCS), extended to their stratified versions, all using SRS without replacement. We have derived the mean, variance, and unbiased variance estimators for these schemes to assess their precision and accuracy. To further enhance precision, we have incorporated ranked-set sampling (RSS) at the secondary and tertiary sampling stages across all schemes and provided mathematical expressions of variances (including their unbiased estimators) for the relevant mean estimators. Through remarks, we have demonstrated that simple random sampling (SRS) with replacement is less efficient than RSS under the same sampling schemes. To validate the performance of RSS against SRS without replacement, we have applied these methodologies to three real-world datasets. The empirical results confirmed that RSS provides greater efficiency than SRS without replacement, as it consistently resulted in lower variance and more precise population mean estimates. These findings directly address the research question of determining which sampling strategies yield the most efficient and precise estimates under 2SCS/stratified two-stage cluster sampling (S2SCS) and 3SCS/stratified three-stage cluster sampling (S3SCS). The study highlights the practical advantage of using RSS in complex survey sampling, particularly when precision and resource efficiency are critical. These results offer valuable insights for survey practitioners, suggesting that adopting RSS can significantly improve the accuracy of estimates while optimizing resource use. Future research can further explore these sampling methods on larger datasets or more intricate multi-stage designs to validate and expand upon the findings.

## Supporting information

S1 Appendix Supplementary Material. This file contain proof of Lemma 3 and 7
(RAR)

S2 Tables Empirical Results of the RE of the Mean Estimators. This file contains RE of the mean estimators under RSS over SRS using 2SCS/3SCS, and S2SCS/S3SCS.
(RAR)

S3 Files Datasets for Population I, II, and III. This file contains datasets supporting the findings of this study.
(RAR)

## Appendix

**Table 1:** List of Abbreviations and Acronyms.

Acronym	Full Term	Reference
2SCS	Two-Stage Cluster Sampling	[8]
3SCS	Two-Stage Cluster Sampling	[8]
S2SCS	Stratified Two-Stage Cluster Sampling	[20]
S3SCS	Stratified Three-Stage Cluster Sampling	[4]
SRS	Simple Random Sampling	[8]
RSS	Ranked-Set Sampling	[18]
2SCS-SRS	2SCS with SRS in Second-Stage of Sampling	[12]
2SCS-RSS	2SCS with RSS in Second-Stage of Sampling	[12]
S2SCS-SRS	S2SCS with SRS in Second-Stage of Sampling	[12]
S2SCS-RSS	S2SCS with RSS in Second-Stage of Sampling	[12]
3SCS-SRS	3SCS with SRS in Third-Stage of Sampling	[12]
3SCS-RSS	3SCS with RSS in Third-Stage of Sampling	[12]
S3SCS-SRS	S3SCS with SRS in Second-Stage of Sampling	[12]
S3SCS-RSS	S3SCS with RSS in Second-Stage of Sampling	[12]
PSUs	Primary Sampling Units	[8]
SSUs	Secondary Sampling Units	[8]
TSUs	Tertiary Sampling Units	[8]
REs	Relative Efficiencies	[8]
BMI	Body Mass Index	[12]
FATA	Federally Administered Tribal Areas	[14]
SES	Socioeconomic Status	[26]
